# The Effect of Exercise Plan Based on FITT Protocol on Primary Dysmenorrhea in Medical Students: A Clinical Trial Study

**Published:** 2019-08-24

**Authors:** Rashid Heidarimoghadam, Elaheh Abdolmaleki, Farideh Kazemi, Seyedeh Zahra Masoumi, Batoul Khodakarami, Younes Mohammadi

**Affiliations:** ^1^Research Center for Health Sciences, School of Public Health, Hamadan University of Medical Sciences, Hamadan, Iran; ^2^Department of Ergonomics, School of Public Health, Hamadan University of Medical Sciences, Hamadan, Iran; ^3^Department of Midwifery, School of Nursing and Midwifery, Hamadan University of Medical Sciences, Hamadan, Iran; ^4^Mother and Child Care Research Center, Hamadan University of Medical Sciences, Hamadan, Iran; ^5^Modeling of Noncommunicable Disease Research Center, Hamadan University of Medical Sciences, Hamadan, Iran; ^6^Department of Epidemiology, School of Public Health, Hamadan University of Medical Sciences, Hamadan, Iran

**Keywords:** Exercise, Dysmenorrhea, Students

## Abstract

**Background:** We aimed to investigate the effects of exercise based on a specific protocol on the severity and duration of primary dysmenorrhea in students residing in dormitories of Hamadan University of Medical Sciences, western Iran in 2017.

**Study design:** Randomized controlled trial study.

**Methods:** Overall, 86 students (43 in the interventional group and 43 in the control group) with mild to moderate dysmenorrhea were enrolled. The exercise based on the FITT protocol (Intensity of exercise, time of exercise, and type of exercise) was implemented for the interventional group in 8 weeks. The McGill Pain scale was used to determine the severity of pain in dysmenorrhea. Duration of pain was calculated in terms of the day. Research data were analyzed using SPSS 20 and the significance level was considered 0.05.

**Results:** The mean dysmenorrhea severity in the first menstrual cycle after the intervention in intervention group was significantly lower than the control group (3.06 (1.78) and 4.74 (2.14), respectively) and in the second menstrual cycle (2.01 (1.54) and 4.61 (2.01) respectively) (*P*<0.001). The mean duration of dysmenorrhea in the first menstrual cycle after the intervention in intervention group was less than the control group (1.29 (0.92) and 2.32 (1.26) respectively) *P*<0.001) and in the second menstrual cycle (0.94 (0.93) and 2.13 (1.24) respectively) *P*<0.001).

**Conclusion:** Sports activities based on a certain and organized protocol could improve dysmenorrhea.

## Introduction

Dysmenorrhea or painful menstruation is a widely common problem of women at the reproductive age^1,2^. The prevalence of dysmenorrhea was 85.7% in Turkey, 73% in Brazil, and 76.1% in Egypt^3^. In Iran, the prevalence of primary dysmenorrhea was 72%^4^. The presence of dysmenorrhea is a disruptive factor in the social, educational and sports life of young women. Despite the fact that the dysmenorrhea does not endanger the real life, it can affect the quality of life, and lead to disability and inefficiency in the case of severe pain. On the other hand, dysmenorrhea can cause psychological problems in some women and lead to isolation and avoidance of participation in various social activities^5^. Most women are now involved in various social scenes, if they do not receive any treatment for this disorder, they will have disabilities for 1-3 d every month.

There are different methods to alleviate symptoms of dysmenorrhea: medication methods such as prostaglandin inhibitors, birth control pills, blockers of calcium channel^7^, non-medication methods such as warming the stomach and back by a hot-water bottle, electric stimulation through skin, acupuncture and compression, aromatherapy, massage therapy, and reflexology^8^, nutritional supplements including the consumption of vitamins E, B, C, calcium and magnesium, elimination of tobacco and coffee, having a good diet with low levels of carbohydrates, and the right amount of protein, vegetables and fruits^9^ and the consumption of medicinal herbs^10^.

Given the role of exercise in the treatment of many diseases over the past two decades, the relationship between physical activity and primary dysmenorrhea has been highly taken into consideration^11^. Reduced dysmenorrhea in women, who exercise, maybe due to the effects of hormonal changes on the uterine connective tissue or increased β-endorphin levels. Beta-endorphin is secreted in blood from hypothalamus neurons in the brain and spinal cord, as well as the pituitary gland. Β-endorphin affects various actions of hypothalamus such as regulation of fertility, heat, cardiovascular actions, respiration, pain perception and mood, and increases the threshold of pain^12^. Women, who are more active and regularly engage in regular exercise, are faced with less physical and mental symptoms than less-active women^13^. Despite the fact that some studies have found no relationship between the level of menstrual pain and primary dysmenorrhea with a level of exercise, there is evidence of the effectiveness of factors such as stretching exercise and mental relaxation^14^. In Tehran, a significant difference was shown duration of pain in dysmenorrhea between the aerobic and non-aerobic exercise groups in adolescent girls^15^.

Accordingly, we decided to investigate the effect of an exercise course based on the ACSM protocol and according to the FITT on the severity and duration of dysmenorrhea. Four-component FITT protocol is a sports protocol proposed by the American College of Sports Medicine (ACSM) referring to four pillars namely the frequency of sports sessions, intensity of exercise, time of exercise, and type of exercise for the experimental group^16^.

## Methods

The present study was a randomized clinical trial on 86 single girl students at Hamadan University of Medical Sciences, Hamadan, western Iran in 2017. The sample size was calculated by considering 5% of the first type error and 90% of the study power and 25% of the sample loss. That way, there were 43 people in each group.

After obtaining the Code of Ethics Committee and obtaining a license from the university, the researcher spoke with students about the goals of the study, and informed written consent forms were collected from the participants.

The inclusion criteria included age ranges from 18 to 24 mild to moderate dysmenorrhea during the last three periods according to the McGill pain scale (1< score≥ 6.6); the absence of known chronic disease or diseases of the reproductive system; regular cycles of every 21 to 35 d; single girl students; no use of any particular chemical or herbal drug affecting dysmenorrhea during the study; no disability; no bans on sports activities due to special medical problems; and no endurance exercises. The exclusion criteria included the reluctance to continue study; the lack of regular trained exercises; and the occurrence of any type of health problem that prevented them from continuing the exercise.

Data collection tools included the demographic data and menstrual status questionnaires as well as McGill pain scale. In order to investigate the validity of demographic data and menstrual status questionnaires, 10 midwifery and physical education professors studied questions and their corrective comments were applied in the case of the research team's approval. McGill pain scale determines the severity of pain and is divided into zero, which means no pain, and 10, which means severe pain; and its validity and reliability were assessed^17,18^. McGill pain scale has been widely used in studies as one of the most usable and reliable measures of pain. The duration of pain per day was also self- reported by participants in the research. In order to determine the sample in this part of the research, the researcher participated in the distribution of the questionnaire by attending the classrooms of the students. At the initial stage of sampling, 258 questionnaires were completed by students through convenience sampling. In other words, based on the results of the questionnaire, only those with mild to moderate dysmenorrhea (1< score≥ 6.6), who chose physical education and sport sciences units 1 and 2, and other criteria for entering the study were included. In general, people who come to study from any college include 11 from the Faculty of Medicine, 23 from the Faculty of Rehabilitation, 13 from the Faculty of Health, 13 from the Faculty of Nursing and Midwifery and 26 from the Faculty of Paramedicine. After determining the subjects, the students who selected the Physical Education Unit 1 in the intervention group and the students who selected the Physical Education Unit 2 were assigned to the control group. And with this way, randomization has also been made.

**Figure 1:**
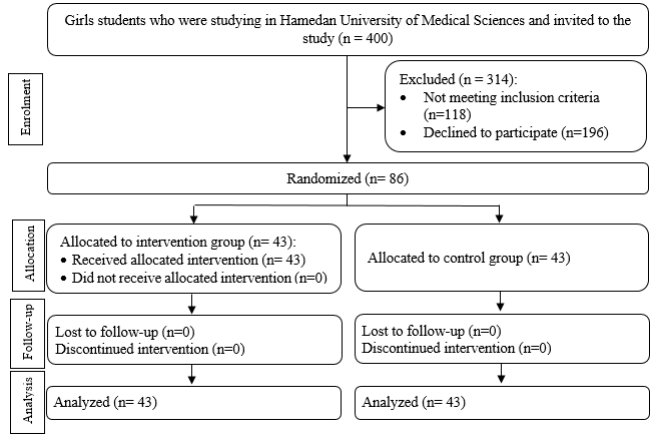
Flowchart contributors to the study

Before the beginning of exercise, the allocation sequence was determined using 4^th^ random blocking. Based on this allocation, students were divided into two groups (Figure 1). Then a demographic data questionnaire including personal information and menstrual status characteristics, a questionnaire for determining the severity of pain based on the McGill pain scale, and the pain duration per day were given to students. The experimental group attended sports sessions for 8 weeks each with 3 sessions (24 sessions) according to designed sport protocol. In each session, a designed program was taught and carried out by a sports instructor with a trained researcher. The way of conducting weekly sports sessions was as follows: individuals in the experimental group participated in their sports classes for 1 out of 3 sessions per week; a session was held in the sports hall with the presence of researcher; samples individually exercised in a session.

Sport version was designed based on the FITT protocol as follows:

F: The frequency of sports program sessions including 8 wk each which with 3 sessions; and each session for a specified period.

I: The maximum heart rate using the Karvonen rule was used to evaluate the exercise intensity according to the following formula:

HRR (Heart Rate Reserve) =%X (HRmax - HR rest) + HR rest

In this formula, the HRR refers to the maximum heart rate; %X is the percentage of exercise intensity; HR max is equal to the maximum heart rate that is equal to age -220 according to the FOX formula.

In this protocol and according to the ACSM guidelines, the exercise intensity should be between 60%- 40% of the maximum heart rate that gradually increases during exercise sessions. In order to evaluate the intensity of exercise, all intervention group individuals were trained to measure heart rate and calculate it before and after exercise and at rest, so that the heart rate was calculated in a minute was by the second and third fingers' touching of the radial artery to find the relative amount of exercise intensity based on the maximum excessive heartbeat on rest at each turn of exercise. The researcher measured heart rates in the present study as was present in sports classes.

T: The exercise time is calculated using the ACSM guidelines. Based on this protocol, training sessions started from 20 min per training session and gradually increased to 47 min during sessions. During each training session, its time increased by a few minutes (2-3 min) until it reached 47 min and then remained at that level.

T: Type of exercise was different during 24 sessions and included a series of aerobic exercises with the main walking base. In each training session, the first 5 min were spent on the warming body and the lasting 5 min for cooling. The basis of sports activity in the experimental group was the endurance of types of exercise, so that during the exercise time, the maximum time was allocated to endurance exercises such as Track and Field along with the set-up, jump rope sport, and jump.

Heart rate was measured once before exercise and once again during exercise to ensure that it increases to the expected level, and once at the end and in the resting phase after exercise to reach heart rate to the baseline. During the study period, the control group did not have any endurance exercise and attended physical education 2 classes only once a week, and carried out group exercises such as volleyball and badminton for 1 h and 30 min.

Finally, the questionnaires of pain severity and duration were completed again by students during the first and second menstrual cycles after the beginning of the intervention. The severity of pain and duration of pain were compared between the two groups. The collected data was analyzed using SPSS 20. Kolmogorov-Smirnov intervention was used to examine the data normality; and Repeated Measure ANOVA, Independent t-test, paired t-test was used for Means comparison. Fisher's intervention with a significance level of *P*<0.05 was used for statistical analysis. The effects of confounding variables such as nutrition and stress have not been studied in this study. Because they have a weak effect on the severity and duration of dysmenorrhea.

## Results

The mean and standard deviation of participants' age was 18.62 (0.65) in the experimental group and 18.67 (0.64) in the control group. The Mean BMI for the experimental group was 21.94 ± 2.83 and in the control group was 21.91 ± 3.24. 27.9% of the subjects in the intervention group and 30.2% in the control group stated that dysmenorrhea sometimes caused them to be absent from school. In the case of absenteeism, 56.25% of those who were always or occasionally forced to absent in the intervention group had one session or half a day and 43.75% had one or more absences. A comparison of two groups in terms of BMI, start age, duration, and intervals of menstruation cycles indicated no significant difference between groups. The majority of participants in both groups had moderate menstrual bleeding and most often had dysmenorrhea in some of their menstruation cycles (Table 1).

**Table 1:** Comparison of demographic and menstrual data of the participants in the research

**Continuous variables**	**Control group, n=43**		**Interventional group, n=43**		***P*** **value**
	**Mean**	**SD**	**Mean**	**SD**	
Age (yr)	18.62	0.65	18.67	0.64	0.757
Body mass index (kg/m^2^)	21.94	2.83	21.91	3.24	0.959
Age of menarche (years)	13.25	1.34	13.13	1.28	0.683
Menstrual bleeding days	5.76	1.34	5.72	1.33	0.872
The interval between two menstruation cycle ( days)	27.90	1.94	27.88	2.02	0.957
**Categorical variables**	**Number**	**Percent**	**Number**	**Percent**	***P*** **value**
Absence from class due to dysmenorrhea					0.228
Yes	4	9.3	5	11.6	
No	27	62.8	25	58.2	
Sometimes	12	27.9	13	30.2	
Amount of bleeding					0.311
Mild	3	7.0	2	4.7	
Moderate	31	72.1	32	74.4	
Severe	9	20.9	9	20.9	
Characteristic of menstrual pain					0.323
All menstruation is painful	2	4.7	3	7.0	
Some menstruation is painful	35	81.3	34	79.0	
Most menstruation is painful	6	14.0	6	14.0	

The mean and standard deviation of pain scores were 4.45 (1.54) in the experimental group before the intervention and 4.43 (1.95) in the control group. The mean comparison indicated that the two groups had no significant difference in terms of the severity of dysmenorrhea. The mean comparison at the first stage of the menstrual cycle after intervention indicated that the mean of dysmenorrhea severity in the experimental group was significantly lower than the control group (3.06 (1.78) and 4.74 (2.14) respectively) *P*<0.001). The mean comparison at the second stage of the menstrual cycle after the start of intervention also indicated that the mean dysmenorrheal severity in the experimental group was less than the control group (2.01 (1.54) and 4.61 (2.01) respectively) and this difference was statistically significant *P*<0.001) (Table 2).

**Table 2:** Comparison of dysmenorrhea severity at different times between two intervention and control groups

**Variables**	**(1) Before** **intervention**		**(2) The 1** ^st^ **menstrual** **cycle after the intervention**		**(3) The 2** ^nd^ **menstrual** **cycle after the intervention**		***P*** **-value**	
	**Mean**	**SD**	**Mean**	**SD**	**Mean**	**SD**	**(1) vs. (2)**	**(1) vs. (3)**
Interventional group	4.54	1.54	3.06	1.78	2.01	1.54	0.001	0.001
Control group	4.43	1.95	4.74	2.14	4.61	2.01	0.040	0.090
*P* value	0.770		0.001		0.001		**-**	**-**

The mean and standard deviation of dysmenorrhea was 2.39 (1.49) in the experimental group before the intervention and 2.18 (1.41) in the control group. The mean comparison indicated that there was no significant difference between the two groups in terms of duration of dysmenorrhea. The mean comparison of the first menstrual cycle after the intervention indicated that the mean dysmenorrhea duration in the experimental group was less than the control group (1.29 (0.92) and 2.32 (1.26) respectively) and this difference was statistically significant (*P*<0.001). The mean comparison ​​at the second stage of the menstrual cycle after the onset of the intervention indicated that the mean dysmenorrhea severity in the experimental group was significantly lower than the control group (0.94 (0.93) and 2.13 (1.24) respectively) *P*<0.001) (Table 3).

**Table 3:** Comparison of duration of dysmenorrhea at different times between two intervention and control groups

**Variables**	**(1) Before** **intervention**		**(2) The 1** ^st^ **menstrual** **cycle after the intervention**		**(3) The 2** ^nd^ **menstrual** **cycle after the intervention**		***P*** **value**	
	**Mean**	**SD**	**Mean**	**SD**	**Mean**	**SD**	**(1) vs. (2)**	**(1) vs. (3)**
Interventional group	2.39	1.49	1.29	0.92	0.94	0.93	0.001	0.001
Control group	2.18	1.41	2.23	1.26	2.13	1.24	0.420	0.420
*P* value	0.510		0.001		0.001		**-**	**-**

## Discussion

The findings of the present study indicated that performing sports activities based on the FITT exercise program led to a reduction in the severity and duration of dysmenorrhea in students. This significant decrease in the duration and severity of dysmenorrhea was consistent with a study with the aim to investigate the effect of Pilates exercise on primary dysmenorrhea. Pilates exercise was carried out for 8 weeks, 3 sessions per week, and each session for 60 minute^19^.

In the present study, the reduction in the severity and duration of dysmenorrhea was consistent with the results of another research^19^. However, the important distinction between the present study and other studies^15,19^ was about the dysmenorrhea and exercise in terms of exercise time per session. The exercise time remained unchanged from the first session of exercise until the final session of exercise time, while in the present study, the initial sessions had a shorter time and their time gradually increased and eventually reached 47 min of activity. Since the research participants were not athletes and had no significant sports activities, the time of physical activity should be slowly increased so that the pain caused by exercise would not be imposed on the body. Results of the present study were inconsistent with another study^20^ on the effect of exercise training on menstruation problems in high school girls during a three-year period. Overall, 39% of the training group and 61% of the control group were suffered from dysmenorrhea^20^. The researchers did not differentiate between primary and secondary dysmenorrhea, and the exercise was carried out for both types of dysmenorrhea. However, people with primary dysmenorrhea and without any organic pelvic or non-pelvic cause were included in the present research.

The intervention group performed walking in 3 consecutive menstrual periods and in the afternoon during the first three days of menstruation in the presence of a trainer for half an hour; and the control group did not perform any activity. In a study, the mean score of pain severity in the experimental group was significantly higher at the end of the study than the pre-study^21^. However, the difference between the present study and another study was the freedom of action to exercise at any time of day. Individuals were free to exercise at any time, while walking was done only in the first three days of menstruation and in the afternoon. There was no difference in the impact of exercises on dysmenorrhea, neither in the morning nor in the afternoon^22^. In another study with the aim to compare the dysmenorrhea status in professional athlete girls and non-athlete girls, professional athlete girls were more faced with symptoms and severity of dysmenorrhea than non-athletic girls; and the finding was inconsistent with results of the present study. The championship sport required longer and more intense exercises that caused excessive fatigue and non-reduction of symptoms in these individuals^23^. One of the reasons for the discrepancy between the present study and another study^23^ was the presence of different samples in both studies as their study samples were professional athlete girls, but samples of the present study were unprofessional students who did not have any long and intense training at the championship level.

Results of the present study were inconsistent with research^15^ who considered three groups namely the aerobic, Kegel and control exercise. The aerobic exercise group trained 3 sessions per week, each session for 45 min; and the Kegel group trained 3 times a day, and each turn for 15 min during 8 wk. Finally, the mean score of pain duration was significantly decreased in terms of hours for both groups of aerobic and Kegel exercise, while the mean pain scores were equal and had no significant changes for the control group before and after the intervention ^15^. A research^24^, with the aim to investigate risk factors in the severity and duration of dysmenorrhea was inconsistent with the results of the present study. In that study, the Menstruation Characteristics Questionnaire used to investigate the causes of dysmenorrhea, and exercise did not affect the severity and duration of dysmenorrhea, but they were affected by weight, fat mass and quality of life^24^. Individuals, evaluated for exercise, were not consistent in terms of the body mass index, while the body weight gain, and especially the increase in fat tissue in central sites of body, struck the balance of steroid hormones. The role of fat tissue is very important in controlling the balance of sex hormones. The fat tissue stores a variety of lipids that can metabolize steroids including androgens and increase the production of prostaglandins after the stimulated endometrium by estrogen and progesterone^25^. Therefore, the homogeneity of BMI as an effective factor in dysmenorrhea is important in both experimental and control groups.

Results of the present study were consistent with another study^26^ to investigate the impact of 8 wk of stretching exercise on the primary dysmenorrhea. In that study, the experimental group carried out selected stretching exercises including 6 types of stretching abdominal, pelvic and groin exercises within 8 wk each which for 3 d, and each day for 2 times, and each time for 10-15 min. At the end of the study, the severity and duration of dysmenorrhea significantly decreased in the experimental group compared with the control group^26^. However, results of another study^27^ were inconsistent with findings of the present the study, where the endurance training did not affect the duration and severity of dysmenorrhea^27^. The difference was probably due to the different types of training and its duration. On the other hand, the low number of participants (15 individuals per exercise group) in their study may affect research results.

The strength of this study over other studies is that we have used a specific exercise program with a specific protocol. In this exercise, the duration and type of exercise performed at each stage are different from the other stages.

The pain assessment scale was the limitation of the study. Since the pain is a mental phenomenon; and its threshold varies in different people, the pain severity cannot be accurately measured in people. On the other hand, various factors such as exercise, socioeconomic status, and nutrition can affect the severity of pain^28-30^. Given the impact of the above-mentioned factors on dysmenorrhea, results of the present study may not be generalized to all young women; hence, such researches are suggested in other regions. And this study was conducted in single and young girls may not be generalized to others.

FITT exercises can be used in young girls to reduce the severity and duration of dysmenorrhea.

## Conclusion

The use of alternative drug therapies has been widely used in the treatment of many diseases; and uncomplicated treatments have found a special place in researchers' studies, especially in the treatment of dysmenorrhea. The training and aerobic exercise significantly reduced the severity and duration of pain. Therefore, this strategy is a good alternative to common drugs such as steroidal anti-inflammatory drugs and oral contraceptives, especially in cases in which these drugs are contraindicated in certain individuals.

## Acknowledgements

The present paper was derived from the student thesis, approved by the Research Council of Hamadan University of Medical Sciences and the Ethics Committee with the code number of IR.UMSHA.REC.1396.312; and also IRCT 201708309014N179 code from the Iranian Registry of Clinical Trials (IRCT). The authors are deeply grateful to all honorable individuals who helped complete the thesis.

## Conflict of interest

The authors declare that there is no conflict of interests

## Funding

This study has been supported financially by the Vice-Chancellor for Research and Technology of Hamadan University of Medical Sciences.

## Highlights

- Aerobic exercise improves pain in dysmenorrhea.
- Exercise based on the FITT protocol is effective in improving the duration of dysmenorrhea.
- Exercise-based on the FITT protocol is effective in improving the severity of dysmenorrhea.
